# Increased risk of A(H1N1)pdm09 influenza infection in UK pig industry workers compared to a general population cohort

**DOI:** 10.1111/irv.12364

**Published:** 2016-01-29

**Authors:** Ellen Fragaszy, David A. Ishola, Ian H. Brown, Joanne Enstone, Jonathan S. Nguyen‐Van‐Tam, Robin Simons, Alexander W. Tucker, Barbara Wieland, Susanna M. Williamson, Andrew C. Hayward, the Flu Watch Group, James L. N. Wood, the Combating Swine Influenza (COSI) Consortium

**Affiliations:** ^1^ Department of Infectious Disease InformaticsFarr Institute of Health Informatics ResearchUniversity College LondonLondonUK; ^2^ Department of Infectious Disease EpidemiologyLondon School of Hygiene and Tropical MedicineLondonUK; ^3^ Immunisation DepartmentPublic Health EnglandLondonUK; ^4^ Animal and Plant Health Agency (formerly Animal Health and Veterinary Laboratories Agency)WeybridgeUK; ^5^ Health Protection and Influenza Research GroupDivision of Epidemiology and Public HealthUniversity of NottinghamNottinghamUK; ^6^ Disease Dynamics UnitDepartment of Veterinary MedicineUniversity of CambridgeCambridgeUK; ^7^ Royal Veterinary CollegeNorth MymmsUK; ^8^ ILRI: International Livestock Research InstituteAddis AbabaEthiopia

**Keywords:** Humans, influenza, occupational exposure, serology, swine, zoonoses

## Abstract

**Background:**

Pigs are mixing vessels for influenza viral reassortment, but the extent of influenza transmission between swine and humans is not well understood.

**Objectives:**

To assess whether occupational exposure to pigs is a risk factor for human infection with human and swine‐adapted influenza viruses.

**Methods:**

UK pig industry workers were frequency‐matched on age, region, sampling month, and gender with a community‐based comparison group from the Flu Watch study. HI assays quantified antibodies for swine and human A(H1) and A(H3) influenza viruses (titres ≥ 40 considered seropositive and indicative of infection). Virus‐specific associations between seropositivity and occupational pig exposure were examined using multivariable regression models adjusted for vaccination. Pigs on the same farms were also tested for seropositivity.

**Results:**

Forty‐two percent of pigs were seropositive to A(H1N1)pdm09. Pig industry workers showed evidence of increased odds of A(H1N1)pdm09 seropositivity compared to the comparison group, albeit with wide confidence intervals (CIs), adjusted odds ratio after accounting for possible cross‐reactivity with other swine A(H1) viruses (aOR) 25·3, 95% CI (1·4–536·3), *P* = 0·028.

**Conclusion:**

The results indicate that A(H1N1)pdm09 virus was common in UK pigs during the pandemic and subsequent period of human A(H1N1)pdm09 circulation, and occupational exposure to pigs was a risk factor for human infection. Influenza immunisation of pig industry workers may reduce transmission and the potential for virus reassortment.

## Introduction

Influenza A viruses can cause significant morbidity and mortality in humans and other animal species and show a high degree of genomic variability and adaptability. They are categorised by subtype based on their main surface glycoproteins, haemagglutinin (HA) and neuraminidase (NA), which determine a range of key properties including antigenicity. Human‐adapted viruses in the past century have been those expressing HA subtypes 1, 2, and 3 and NA subtypes 1 and 2. Since 1968, only the A(H1N1) and A(H3N2) subtypes have circulated widely in humans.^1^ Observations over the past 40–50 years have documented subtypes of viruses A(H1N1), A(H1N2) and A(H3N2) circulating in pigs worldwide and strain variations between Europe, North America and Asia have been noted.^2^ In the UK, A(H1N2) was the most commonly observed swine subtype in a large pig serosurvey conducted between 2008–2009.^3^ Between 1998 and 2009, an avian‐like H1N1 strain most commonly and an H1N2 strain were regularly detected in UK pigs.^4^, ^5^ The A(H1N1)pdm09 virus was detected in pig herds from autumn 2009,^6^ although it may have been first transmitted to pigs from humans several months earlier.^7^

Influenza viruses bind to host cell surface receptors with a terminal sialic acid (SA), different versions of which are present in different animal species forming the basis of host‐strain specificity.^8^, ^9^ Avian strains preferentially bind to SA α2,3‐Gal (prevalent in avian species) while human virus strains require SA α2,6‐Gal receptors (dominant in humans). The relatively poor fit of avian viruses to human and other non‐avian hosts is thought to limit the potential emergence of novel strains^10^ Pigs (and many other species) express both types of receptors such that they could be potentially susceptible to both avian and human viruses.

Co‐infection of a single host with two different strains of the influenza virus provides an opportunity for genetic reassortment (rearrangements and altered combinations of genome segments), which could lead to sudden and marked changes (antigenic shift) and the emergence of novel strains or subtypes expressing new surface antigen proteins that the host might have little or no immunity against. Should the newly acquired properties of such a novel strain make it transmissible in humans, then it would have the potential to start a pandemic. Pigs are a particularly important species in this regard as the occurrence of both types of SA receptors permits binding of human and avian influenza viruses making them an efficient ‘mixing vessel'.^11^, ^12^, ^13^, ^14^ Interspecies transmission (in both directions) of swine and human influenza viruses is well recognised, evidenced by the isolation of human influenza virus in swine^15^, ^16^ and evidence of swine influenza virus (SIV) infection in people with close occupational^17^, ^18^, ^19^ and/or residential proximity to pigs^20^, ^21^; or prolonged exposure at an agricultural fair^22^. Transmission between pigs and bird species is exemplified by various reports of isolation of SIV from turkeys.^23^, ^24^, ^25^ The 2009 pandemic virus A(H1N1)pdm09 comprised genetic components from the swine‐adapted North American triple reassortant H3N2 viruses and a Eurasian swine virus.^26^

There is an increasing need for monitoring transmission between pigs and humans, but data on the extent of such transmission events remains limited. Previous studies attempting to assess serological evidence of swine influenza in people with occupational exposure to pigs all recruited their non‐pig‐exposed comparison groups from restricted groups such as blood donors,^18^, ^27^, ^28^, ^29^ students, teachers, or university or hospital personnel,^30^, ^31^, ^32^ or in some cases, they used serum bank samples.^17^, ^33^ This study focused on assessing SIV infection in pig industry workers in England during the emergence of A(H1N1)pdm09 virus. Serological data on SIV infection in pig veterinarians and pig farm workers was compared with a sample from the general population, and related to serology from sampled pigs in contact with the pig farm workers.

## Methods

### Recruitment and specimen collection

We recruited pig industry workers including pig farm workers and specialist pig veterinarians (each veterinarian typically attended a number of different farms across an area, and some also worked in other settings such as abattoirs). Pig veterinarians were recruited at November 2009 and May 2010 meetings of the Pig Veterinary Society, a species specialist group of the British Veterinary Association. Pig farm workers were recruited from 17 farms in September–December 2010 from a large group of farrow‐to‐finish pig farms that participated in a related study of SIV infection in English pigs.^3^ Farms came from two main clusters in North Yorkshire and East Anglia, both regions with higher densities of the pig population.^34^ Farm owners were first asked for permission to approach their staff, including everyone with direct pig contact such as farm hands, on‐site managers, and field maintenance workers. At the farms where owners granted permission, pig farm workers were invited to join the study. At the same time blood samples were collected from pigs from each of the worker's farms.

Participants from the concurrent Flu Watch study – a community‐level, household‐based cohort study of influenza in England^35^ – formed the population comparison group. Flu Watch participants were frequency‐matched to pig industry workers on age group, geographic region, calendar month of blood sample, and gender (in decreasing priority order).

All participants gave individual written informed consent, and completed a questionnaire including information on demographic characteristics and their history of influenza vaccination for that season (2009 for pig veterinarians or 2010 for pig farm workers). Blood samples were collected from all participants for serological analysis.

To examine the association between SIV infection among pig farm workers and SIV infection among the pigs they worked with, blood specimens were obtained from a sample of pigs on their farms as part of the aforementioned SIV infection study.^3^ Blood specimens were taken from pigs during the same season as the pig farm workers (autumn 2010).

### Influenza virus panel and laboratory methods

Serum samples from pig industry workers and the Flu Watch population comparison group were tested for the presence of antibodies using an AHVLA standard panel of SIVs representative of contemporary viruses detected through routine SIV surveillance in UK pigs, and known human viruses^5^ (see Table S1). The SIVs in the panel were A/sw/England/117316/86 classical H1N1 (classical swine H1N1); A/sw/England/195852/92 avian‐like H1N1 (swine avian‐like H1N1); A/sw/England/163266/87 H3N2 (swine H3N2 87); and A/sw/England/438207/94 H1N2 [swine H1N2]. The human viruses were A/England/195/09 pH1N1 [A(H1N1)pdm09]; A/Brisbane/59/07 H1N1 (H1N1 07); and A/Perth/16/09 H3N2 (H3N2 Perth). Standard haemagglutination inhibition (HI) assays^36^ were used. A reciprocal antibody titre of ≥40 (1:40 from serial dilution) was considered seropositive and taken as indicative of putative previous infection with the corresponding virus in humans.

Sera from unvaccinated pigs were tested for a smaller subset of viruses [classical swine H1N1, swine H1N2, swine H3N2 87, swine avian‐like H1N1, and A(H1N1)pdm09]. It is recognised that in HI tests with pig sera, the profile against the range of viruses used needs to be analysed and interpreted with care, as homosubtypic cross‐reactive antibodies to the HA may be detected without inferring exposure to a particular strain. Difficulties in swine HI serology interpretation can be compounded further by anti NA (especially N2) antibodies interfering in the HI test. Our approach was to evaluate the titres to determine those of the greatest magnitude correlating with the most probable virus subtype an individual animal had been exposed to. Within a subtype, if the highest titre was ≥40 then the pig was considered seropositive for that strain. If two strains within a subtype shared the highest titre (≥40) then the pig was considered seropositive for both; although it should also be noted that a single animal may have been exposed to more than one influenza virus.

Given that most farms had 12–16 pigs tested, we considered a farm positive for a given strain if it had at least three pigs seropositive for that strain.

### Statistical analysis

We explored whether occupational exposure to pigs was associated with infection with each virus strain through univariable analysis using chi‐square (χ^2^) and Fisher's exact tests. We then built separate multivariable logistic regression models for each virus strain to estimate the association of occupational exposure to pigs and infection. These models accounted for clustering for repeated measurements as some participants contributed more than one sample from different time periods. In each model we investigated the potential confounding effects of vaccination status, age, season (winter 2009, spring 2010, autumn/winter 2010), geographic region and gender. A variable was retained in the model if it was associated with occupational pig exposure, associated with infection, and either independently predicted the outcome or else made an appreciable difference on the effect of occupational pig exposure on infection. We hypothesised, *a priori*, that the season of the blood sample may modify the effect of occupational pig exposure on infection and this was explored by testing for interaction terms in the models.

Where an influenza strain was found to be associated with occupational pig exposure, we investigated the possibility of cross‐reactivity between that strain and other swine viruses sharing the same haemagglutinin using cross‐tabulations with chi‐square or Fisher's exact test as appropriate. Where there was evidence of association these strains were forced into regression models to account for possible cross‐reactivity.

We conducted subanalyses among pig veterinarians providing more than one blood sample (November 2009 and May 2010) to calculate the risk of seroconversion to each virus strain, as determined by a fourfold rise in antibody titre.

In a series of subanalyses (one for each strain of SIV tested in both pigs and humans), we explored whether pig farm workers' SIV seropositivity status was associated with the positivity status of their farm's pig herd using chi‐square and Fisher's exact tests.

### Ethics

This study was approved by the Cambridgeshire‐1 Research Ethics Committee (REC) Reference 10/H0304/4. The Flu Watch study, from which the population comparison group was drawn, was approved by the Oxfordshire REC Reference 06/Q104/103. Participants received full information about the study and if interested and eligible, they were enrolled after providing fully informed written consent.

## Results

### Participants and blood samples

The characteristics of participants and number of blood samples are described in Table 1. A total of 26 pig veterinarians participated in the study, providing 42 separate blood samples, with 16 veterinarians contributing two samples (one from November 2009 and one from May 2010). An additional 29 pig farmers from 17 different pig farms participated in the study, each contributing one blood sample. A total of 68 Flu Watch participants provided 71 blood samples which were frequency‐matched to the samples from the pig industry workers as described in the methods. Sixty‐five of the Flu Watch participants contributed only one blood sample but three contributed two blood samples from two of the three possible seasons (winter 2009, spring 2010 or winter 2010). Most pig industry workers were male. The median age for pig industry workers and the frequency‐matched Flu Watch participants was 44 and 47 respectively. At the time the blood sample was taken, 93% of participants were unvaccinated. Only five Flu Watch participants and four pig farmers had received the currently available pandemic vaccine.

**Table 1:** Participant characteristics and numbers of samples

Participant characteristics	Flu watch				Pig industry workers			
	No. people (*N* = 68)^a^	%	No. blood samples (*N* = 71)^a^	%	No. people (*N* = 55)^a^	%	No. blood samples (*N* = 71)^a^	%
Age group								
<45	29	43	30	42	28	51	36	51
45–64	34	50	36	51	23	42	31	44
65+	5	7	5	7	4	7	4	6
Gender								
Male	55	81	57	80	45	82	57	80
Female	13	19	14	20	10	18	14	20
Region in England								
East Midlands	30	44	31	44	21	38	31	44
North East	17	25	18	25	16	29	18	25
London and SE	14	21	14	20	11	20	14	20
West	7	10	8	11	7	13	8	11
Influenza vaccination								
No	63	93	65	92	51	93	67	94
Yes	5	7	6	8	4	7	4	6
Pig industry worker type								
Veterinarian	N/A	–	N/A	–	26	47	42	59
Farmer	N/A	–	N/A	–	29	53	29	41

a Number of people differ from number of blood samples as some individuals provide blood samples for more than one season.

### Risk of infection in relation to occupational exposure to pigs

In the univariable analysis (Table 2), there was evidence that antibodies to three out of the eight influenza strains were more common in pig industry workers than the population comparison group: A(H1N1)pdm09 (23% versus 4%, *P *=* *0·001); swine H1N2 (24% versus 11%, *P *=* *0·047) and H3N2 Perth (37% versus 20%, *P *=* *0·025).

**Table 2:** Crude risk and adjusted odds of influenza infection comparing pig industry workers to a sample from a general population cohort (flu watch)

Typical host	Strain	Univariable analysis							Multivariable regression analysis		
		Flu watch			Pig industry worker			*P*‐value			
		*N*	No. positive (no. of these who were vaccinated)	% Pos	*N*	No. positive (no. of these who were vaccinated)	% Pos	Chi^2^	Adjusted OR (95% CI) pig industry worker versus flu watch	*P*‐value	Model covariates^c^
Swine	A/sw/England/117316/86 classical H1N1 (classical swine H1N1)	71	3 (2)	4	71	7 (3)	10	0·326^b^	4·7 (0·81–27·90)	0·085	Vaccination
	A/sw/England/195852/92 avian‐like H1N1 (swine avian‐like H1N1)	71	0 (0)	0	71	3 (1)	4	0·245^b^	—		
	A/sw/England/163266/87 H3N2^a^ (swine H3N2 87)	53	34 (3)	64	53	28 (4)	53	0·237	0·76 (0·33–1·77)	0·522	Sex, season, age group
	A/sw/England/438207/94 H1N2 (swine H1N2)	71	8 (2)	11	71	17 (4)	24	0·047	4·32 (1·39–13·46)	0·012	Vaccination, season
	*Controlled for possible cross‐reactivity* d								3·91 (1·19–12·87),	0·025	As above+^d^
Swine and human	A/England/195/09 pH1N1 [A(H1N1)pdm09]	71	3 (2)	4	71	16 (4)	23	0·002^b^	20·44 (2·24–186·40)	0·007	Vaccination
	*Controlled for possible cross‐reactivity* e								15·11 (1·64–139·75)	0·017	As above +^e^
Human	A/Brisbane/59/07 H1N1 (H1N1 07)	71	15 (4)	21	71	15 (3)	21	1	1·11 (0·45–2·74)	0·22	Vaccination
	A/Perth/16/09 H3N2 (H3N2 Perth)	71	14 (3)	20	71	26 (4)	37	0·025	3·77 (1·52–9·35)	0·004	Vaccination, season
	*Controlled for possible cross‐reactivity* f	37	9 (2)	24	30	5 (0)	17	0·443	4·22 (1·28–13·94)	0·018	As above+^f^

a Limited to 106 samples with H3N2 87 readings.

b Fisher's exact test *P*‐value.

c Possible covariates include age group, gender, region, season and vaccination status.

d Controlled for seropositivity to for classical swine H1N1, swine avian‐like H1N1, A(H1N1)pdm09.

e Controlled for seropositivity to classical swine H1N1, swine avian‐like H1N1 or swine H1N2.

f Controlled for seropositivity to H3N2 87 and swine H1N2.

There was no evidence of swine avian‐like H1N1 antibodies in the population comparison group in contrast to three seropositive pig industry workers (4%). Although 10% of pig industry workers and 4% of the comparison group had antibodies to classical swine H1N1, these reactions were most probably due to cross‐reactive antibodies from an A(H1N1)pdm09 infection as the classical swine H1N1 strain had not circulated in the UK for decades and 70% of those seropositive for the virus were also seropositive for A(H1N1)pdm09. Antibodies to swine A(H1N2 or H3N2) strains were relatively common in both groups (range 11–64%).

In the multivariable analysis (Table 2), after adjusting for confounders, there was strong evidence that pig industry workers had elevated odds of A(H1N1)pdm09 seropositivity [adjusted odds ratio (aOR) = 20·4, 95% confidence interval (CI) (2·2–186·4), *P* = 0·007] compared to the Flu watch comparator population. We found strong evidence that A(H1N1)pdm09 seropositivity in humans was associated with seropositivity to swine H1N2 (*P* = 0·003), classical swine H1N1 (*P* < 0·001) and swine avian‐like H1N1 (*P* = 0·002). The association between A(H1N1)pdm09 seropositivity and occupational swine exposure remained strong after controlling for the possible effect of cross‐reactivity with these strains [aOR = 15·1, 95% CI (1·6–140), *P* = 0·017].

Pig industry workers had an increased odds of swine H1N2 seropositivity [aOR = 4·3 (95% CI 1·4–13·5), *P* = 0·012] compared to the population group. There was strong evidence that seropositivity was associated with A(H1N1)pdm09 (*P* = 0·003) and classical swine H1N1 (*P* < 0·001) but less evidence of an association with avian‐like swine H1N1 (*P* = 0·080). The odds ratio remained elevated after controlling for the possible effect of cross‐reactivity with these strains [aOR = 3·9, 95% CI (1·2–12·9), *P* = 0·025].

Pig industry workers also had an increased odds of H3N2 Perth seropositivity [aOR = 3·8, 95% CI (1·5–9·4), *P* = 0·004] compared to Flu Watch participants. We found limited evidence of an association between the Perth and the swine H3N2 87 strain (*P* = 0·087) and strong association with the swine H1N2 strain (*P* = 0·001). After controlling for possible cross‐reactivity with these strains the odds ratio remained elevated [aOR = 4·2, 95% CI (1·35–13·9), *P* = 0·018]. As H3N2 influenza strains have not circulated in UK pigs since 1997, we examined this association separately in those who were aged <18 years in 1997 (<30 years at the time of the study) and those who were aged over 18 years in 1997 (30 years or over at the time of the study). We found no association between pig worker occupation and H3N2 Perth in the younger age group [aOR 0·5, 95% CI (0·0–16·1), *P* = 0·696], but a strong association in the older group [aOR 6·0, 95% CI (1·5–22·9), *P* = 0·01].

There was no evidence to suggest that occupational pig exposure increased the odds of seropositivity to the other influenza strains tested.

There was no evidence that season modified the association between occupational exposure to pigs and seropositivity to any of the remaining viruses tested.

### Seroconversion among pig veterinarians

Five of the 16 pig veterinarians with repeat samples seroconverted to one or more strains tested and none had received influenza vaccination between blood samples. One veterinarian seroconverted to four different viruses [human H1N1 07, A(H1N1)pdm09 and swine H3N2 87] while another veterinarian seroconverted to both human H1N1 07 and A(H1N1)pdm09. The other three veterinarians either converted to human H3N2 Perth or swine H1N2.

### Pig serology and farm‐level seroprevalence

Serology results for pigs were linked for 14 of 17 farms (corresponding to 214 pigs in contact with 25 pig farm workers). Pig‐ and Farm‐level seroprevalence is reported in Table 3. Farm‐level positivity for a strain meant at least three seropositive pigs for that strain on the farm. After accounting for possible homosubtypic cross‐reactive antibodies in the three A(H1) strains tested in pigs, we found that 41% of pigs were seropositive to A(H1N1)pdm09 and 79% of farms were considered positive for the strain. In contrast, only 3–5% of pigs were positive for classical swine H1N1, swine avian‐like H1N1 and swine H3N2 87. No farms were positive for either swine H1N1 strains and only one farm was positive for swine H3N2 87.

**Table 3:** Seroprevalence of SIV infection among pigs on farms linked to one or more pig farmers

Farm	No. pigs tested	Classical swine H1N1			Swine H3N2 87			Swine avian‐like H1N1			A(H1N1)pdm09		
		No. positive pigs	% Positive pigs	Farm considered positive^a^	No. positive pigs	% Positive pigs	Farm considered positive^a^	No. positive pigs	% Positive pigs	Farm considered positive^a^	No. positive pigs	% Positive pigs	Farm considered positive^a^
1	16	0	0	No	0	0	No	0	0	No	8	50	Yes
2	12	2	17	No	0	0	No	1	8	No	3	25	Yes
3	16	0	0	No	0	0	No	0	0	No	8	50	Yes
4	12	0	0	No	0	0	No	0	0	No	5	42	Yes
5	12	0	0	No	8	67	Yes	0	0	No	7	58	Yes
6	16	0	0	No	0	0	No	0	0	No	15	94	Yes
7	10	0	0	No	0	0	No	0	0	No	0	0	No
8	16	0	0	No	1	6	No	2	13	No	4	25	Yes
9	12	2	17	No	0	0	No	0	0	No	0	0	No
10	16	1	6	No	0	0	No	2	13	No	3	19	Yes
11	12	0	0	No	0	0	No	1	8	No	1	8	No
12	16	2	13	No	0	0	No	0	0	No	10	63	Yes
13	16	0	0	No	0	0	No	0	0	No	8	50	Yes
14	32	2	6	No	1	3	No	0	0	No	15	47	Yes
Total	214	9	4	0 of 14	10	5	1 of 14	6	3	0 of 14	87	41	11 of 14

a Farms were considered positive if three or more animals in that herd tested positive (titres ≥ 40 and highest titre within HA subtype).

### Farm‐level seroprevalence and human infection

There was no evidence of an association between farm positivity and risk of infection among pig farm workers for any of the strains tested. All pig farm workers infected with the pandemic virus worked on a farm positive for the same strain. No pig farm workers were infected with swine avian‐like H1N1 (Table 4).

**Table 4:** Association between pig farm workers' infection status and the positivity status of the pig herd they work with

Strain	Pig farmers working on positive farm	Pig farm workers				
		Seronegative		Seropositive		*P*‐value^a^
		*N*	Column %	*N*	Column %	
Classical swine H1N1	No	23	100	2	100	n/a
	Yes	0	0	0	0	
Swine H3N2 87	No	12	100	10	77	0·220
	Yes	0	0	3	23	
Swine avian‐like H1N1	No	25	100	0	n/a	n/a
	Yes	0	0	0	n/a	
A(H1N1)pdm09	No	6	29	0	0	0·540
	Yes	15	71	4	100	

a Fisher's exact test.

## Discussion

This study improves our understanding of swine influenza transmission to humans, by comparing the serological evidence of SIV seropositivity in pig industry workers in England with a general population‐based comparison group at the time of the A(H1N1)pdm09 influenza pandemic.

The key finding is that, in the period of this study, pig industry workers had increased odds of influenza A(H1N1)pdm09 seropositivity compared to the general population. Evidence of the association remained after controlling for seropositivity to other swine H1 viruses, and is thus unlikely to be the result of cross‐reactivity. We also found evidence that pig industry workers had elevated odds of swine H1N2 and H3N2 Perth seropositivity which remained after controlling for seropositivity to other measured, potentially cross‐reactive strains.

The increased risk of A(H1N1)pdm09 in pig industry workers is compatible with the concurrent emergence of infection with A(H1N1)pdm09 in pigs in England, which was first observed in November 2009^6^ and confirmed by the serological results in our study. As there was minimal trade of live pigs between North America and Europe during the period of the study and no reports of the pandemic strain in European pigs prior to human cases,^37^ it is likely that pigs were initially infected by humans during the early stages of the 2009 pandemic, and infection then transmitted efficiently within and between pig herds but also through reverse zoonoses events following contact of pigs with infected humans. Phylogenetic analysis has subsequently demonstrated that H1N1pdm2009 has been repeatedly transmitted from humans to swine since the pandemic.^38^ Pig industry workers naïve to A(H1N1)pdm09 would be susceptible to zoonotic infection from pig herds undergoing active infection, with exposure to, sometimes large, groups of pigs simultaneously undergoing acute infection and shedding virus favouring transmission from pigs to pig industry workers. Further bidirectional transmission may have led to an amplification effect leading to high levels of infection in both pigs and pig industry workers. This is important in that it shows that dense populations of pigs can serve as an amplifying reservoir for influenza virus, increasing the risk of novel virus transmission to both pigs and to man. This has been illustrated during an outbreak of H1N1pdm2009 on a research farm in Canada^39^ and explored in mathematical models of the potential amplifying impact of such bidirectional transmission.^40^

Our findings overall are consistent with other work identifying increased risk of influenza A(H1N1)pdm09 in pig industry workers compared to others without occupational pig exposure. However, they could not exclude cross‐reactivity between other SIVs and influenza A(H1N1)pdm09 as the cause;^29^, ^33^ and others have reported no increased risk.^30^, ^41^ We found evidence of an increased risk of the A(H1N1)pdm09 strain which is known to affect both pigs and humans in pig industry workers even after controlling for potential cross‐reactivity and the effect was also not due to confounding by age, region, and time of sample or vaccination.

With regard to other SIV strains other than A(H1N1)pdm09, previous studies found an increased risk of seropositivity to at least one SIV in pig workers, including H1N1,^17^, ^18^, ^27^, ^29^, ^31^, ^33^, ^41^, ^42^, ^43^ H1N2^18^, ^28^, ^31^ and H3N2^28^, ^29^, ^30^, ^44^ strains. In our study, we found increased risk of seropositivity both to swine H1N2 and H3N2 Perth. This increased risk remained after controlling for potential cross‐reactivity with measured strains. The increased risk of seropositivity to swine H1N2 is consistent with occupational exposure. The increased risk of H3N2 Perth (a human strain) was not explained by cross‐reactivity to swine H3N2 87 or swine H1N2. Others have found H3N2 Perth strain assays to cross‐react strongly with swine H3N2.^45^ Thus it is plausible that the increased risk of H3N2 seropositivity in pig workers in our study was due to cross‐reactivity with an unmeasured H3N2 swine strain. This is further supported by the fact that the association was only found in those aged 30 years or more who would have been of working age when H3N2 strains last circulated widely in UK swine in 1997. A further unexpected finding was the high levels of antibodies to swine H3N2 87 in the general population and in pig workers. This could also be explained through cross‐reactivity with human H3N2 strains.

In contrast to all the previous studies which compared pig workers to highly selective groups, our work has the advantage of using a general population comparison group, frequency‐matched for age, region, month of bleed and gender. Although we could not exclude pig exposure in the control group such exposure is likely to be rare in the general UK population. The work is challenged by limited ability of laboratory tests to exclude cross‐reactivity between all viral strains, a common issue with studies of this nature. Future work using microneutralisation assays would reduce uncertainty over cross‐reactivity.

It is generally considered that influenza virus reassortment with significant pandemic potential is most likely to occur in developing country ‘hotspots'^46^, where the demographic, cultural and economic circumstances and animal husbandry practices together result in settings of dense overlaps between humans and animal populations and opportunities for cross‐species transmission. However, given our findings, and observations of new reassortant strains elsewhere in Europe^47^, ^48^, there should be no assumption that reassortment with possible zoonotic risk could not also occur in industrialised settings.

The study was unable to examine whether there was also an increased risk of clinical disease in pig industry workers, but the work suggests the need for coordinated enhanced surveillance in both pigs and pig industry workers. Observations from this study also offer strong supporting evidence that pig industry workers should be among the occupational groups offered annual seasonal influenza vaccination. Preventing influenza infection in people who work with pigs would seem to be a logical option to minimise the risk of transmission of human variants into pigs, and by extension to reduce the possibilities for reassortment in pigs.

## Financial support

This work was supported by joint funding from the Biotechnology and Biological Sciences Research Council (BBSRC), the Medical Research Council (MRC), and the Wellcome Trust (WT) [(BBSRC/MRC/WT) BB/H014306/1; (MRC/WT) MC_U122785833; (MRC) G0800767 and G0600511]; Alborada Trust (to J.L.N.W.); the RAPIDD programme of the Science & Technology Directorate (to J.L.N.W.); US Department of Homeland Security (to J.L.N.W.); and the Fogarty International Center at the National Institutes of Health (to J.L.N.W.). DAI is supported by a fellowship from the National Institute for Health Research (NIHR) (PDF‐2012‐05‐305) (this research is independent and the views expressed in this publication are those of the authors and not necessarily those of the Department of Health or NIHR).

## Competing interest

Prof. Nguyen‐Van‐Tam reports grants from GlaxoSmithKline, grants from F. Hoffmann‐La Roche, and non‐financial support from the European Scientific Working Group of Influenza, outside the submitted work; and over one decade ago, he was employed by SmithKline Beecham (now a part of GlaxoSmithKline – manufacturer of zanamivir and influenza vaccine), from 2000 to 2001; by Roche Products Ltd (manufacturer of oseltamivir) from 2001 to 2002; and by Aventis Pasteur MSD (now Sanofi Pasteur MSD – manufacturer/distributor of influenza vaccine) from 2002 to 2004. He holds no shares, share options or pension rights in any of these companies. He performed paid consultancy for several influenza vaccine manufacturers in the period 2008 to 2010. His brother was an employee of GlaxoSmithKline until August 2015, but did not work in an influenza‐related field. DAI reports participation in public sector research that received non‐financial support from Novartis and GlaxoSmithKline, outside the submitted work. Authors EBF, IAH, JE, RS, AWT, BW, SW, ACH and JW have no competing interests.

JSN‐V‐T is Editor‐in‐Chief of Influenza and Other Respiratory Viruses; however he played no role whatsoever in the editorial process for this paper, including decisions to send the manuscript for independent peer‐review or about final acceptance of a revised version. All of the above functions were handled alone and independently by Dr Alan Hampson, Senior Editor (formerly Editor‐in‐Chief).

## Addendum

James Wood (JW) and Ian H Brown (IAH) were overall PIs on the grants funding the work. Andrew C Hayward (ACH) was PI of the Flu Watch study. JW, IHB and ACH contributed to the conception and design of the studies. Barbara Weiland (BW) contributed to the planning of the pig farmer study, coordinated recruitment of pig farms, planned and oversaw field work on pig farms. Joanne Enstone (JE) collected data and samples from pig farmers. Susanna Williamson (SW) and Alexander W Tucker (AWT) suggested involvement of pig veterinarians in the study, organised their recruitment and sampling, and contributed to questionnaire development and other aspects of the study. David A Ishola (DAI) contributed to study planning and design, and collected data and samples from pig veterinarians. IHB led the serological analysis and interpretation. Ellen B Fragaszy (EBF) contributed to Flu Watch and pig veterinarian data collection, led the Flu Watch data management and designed and conducted the statistical analysis. Robin Simons (RS) contributed to the design and interpretation of the statistical analyses. DAI and EBF wrote the manuscript with contributions from ACH, JW, IHB, JSN‐VT‐, SW, RS and BW. All authors made contributions to manuscript review and approved the final version.

## Supporting information


**Table S1.** Description of influenza Strain names, typical host and whether antibodies were tested in humans and pigs.
