# Supplementary material for: Increased risk of A(H1N1)pdm09 influenza infection in UK pig industry workers compared to a general population cohort
Source: Influenza Other Respir Viruses. 2016 Jan 29;10(4):291–300. doi: 10.1111/irv.12364 (PMC4910179; doi:10.1111/irv.12364)
Supplement: Supplementary file 1 — Table S1. Description of influenza Strain names, typical host and whether antibodies were tested in humans and pigs. [file IRV-10-291-s001.docx]

**Supplemental Appendix**

Fragaszy and Ishola et al., Increased risk of A(H1N1)pdm09 influenza infection in UK pig industry workers compared to a general population cohort, *Influenza and Other Respiratory Viruses, 2015*

**Correspondence:**

Dr. Andrew Hayward

Department of Infectious Disease Informatics

Farr Institute of Health Informatics Research

University College London

222 Euston Road

London NW1 2DA

United Kingdom

Email: [a.hayward@ucl.ac.uk](mailto:a.hayward@ucl.ac.uk)

**COmbating Swine Influenza (COSI) consortium:**

(i) Animal Health and Veterinary Laboratories Agency, Addlestone, UK: Ian H. Brown, Sharon M. Brookes, Alasdair J.C. Cook, Susanna Williamson; (ii) Cambridge Infectious Diseases Consortium, Department of Veterinary Medicine, Cambridge, UK: James L.N. Wood, Alexander W. Tucker, Julia R. Gog, Roberto A. Saenz, Pablo Murcia; (iii) Imperial College London, London, UK: Wendy S. Barclay, Christl A. Donnelly; (iv) Wellcome Trust Sanger Institute, Cambridge, UK: Paul Kellam; Royal Veterinary College, Hatfield, UK: Barbara Wieland; (v) National Institute for Medical Research, London, UK: John McCauley; (vi) Scottish Agricultural College, Edinburgh, UK: George Gunn; (vii) Centre for Infectious Disease, University of Edinburgh, Edinburgh, UK: Andrew Leigh Brown, Samantha J. Lycett, Mark Woolhouse; (viii) Department of Zoology, Oxford University, Oxford, UK: Oliver Pybus; (ix) University College London/Flu Watch, London, UK: Andrew C. Hayward; (x) The Roslin Institute and R(D)SVS, University of Edinburgh, Edinburgh, UK: Alan L. Archibald; (xi) Institute for Animal Health, Compton near Newbury, UK: Bryan Charleston, Eric A. Lefevre; (xii) Division of Veterinary Pathology, University of Bristol, Bristol, UK: Mick Bailey, Charlotte F. Inman; (xiii) School of Veterinary Medicine and Science, University of Nottingham, Nottingham, UK: Kin Chow Chang, Stephen P. Dunham.

**Flu Watch Group**

**The Flu Watch Group consists of all those who have played a substantive role in study design and/or data collection, analysis and interpretation.**

Bermingham A (1), Cooke M (2), Copas A (2), Edmunds J (3), Fragaszy E (2,3), Ferguson N (4), Goonetilleke N (5), Harvey G (2), Hayward AC (2), Johnson AM (2), Kovar J (2), Lettley L (6), Lim M (2), Mazo D- visiting scientist (2), McMichael A (5), Millett ERC (2, 3), Nazareth I (7), Nguyen-Van-Tam JS (8), Pebody R (2, 9), Wang L (5), Watson JM (2, 10), Wurie F (2), Zambon M (1)

1. Respiratory Virus Unit, Centre for Infections, Public Health England
2. UCL Farr Institute of Health Informatics Research
3. London School of Hygiene and Tropical Medicine, Department of Infectious Disease Epidemiology
4. Imperial College London
5. University of Oxford - Weatherall Institute of Molecular Medicine
6. Medical Research Council General Practice Research Framework.
7. UCL Department of Primary Care and Population Health
8. Health Protection and Influenza Research Group, Division of Epidemiology and Public Health, University of Nottingham, UK
9. Public Health England – Respiratory Diseases Department

10) Department of Health

**Supplemental Table**

**Table 1 – Description of influenza Strain names, typical host and whether antibodies were tested in humans and pigs**

| **Typical Host** | **Virus** | **Abbreviated name** | **Antibodies tested** | |
| --- | --- | --- | --- | --- |
|  |  |  | **Humans** | **Pigs** |
| Swine | A/sw/England/117316/86 classical H1N1 | classical swine H1N1 | x | x |
| Swine | A/sw/England/195852/92 avian-like H1N1 | swine avian-like H1N1 | x | x |
| Swine | A/sw/England/163266/87 H3N2 | swine H3N2 87 | x | x |
| Swine | A/sw/England/438207/94 H1N2 | swine H1N2 | x |  |
| Swine & Human | A/England/195/09 pH1N1 | A(H1N1)pdm09 | x | x |
| Human | A/Brisbane/59/07 H1N1 | H1N1 07 | x |  |
| Human | A/Perth/16/09 H3N2 | H3N2 Perth | x |  |
